# Streptococcal Toxic Shock Syndrome in a Patient with a Phlycten 

**DOI:** 10.29252/beat-070415

**Published:** 2019-10

**Authors:** Neuza Soares, Ana Rita Parente, Clara Gomes, Rodrigo Pimentel

**Affiliations:** 1 *Internal Medicine Service, Centro Hospitalar Universitário de São João, Porto, Portugal*; 2 *Internal Medicine Service, Centro Hospitalar das Caldas da Rainha, Caldas da Rainha, Portugal*; 3 *Intensive Medicine Service, Centro Hospitalar Universitário de São João, Porto, Portugal *

**Keywords:** Streptococcal Toxic Shock Syndrome, Phlycten, Fasciotomy

## Introduction

**Fig. 1.**
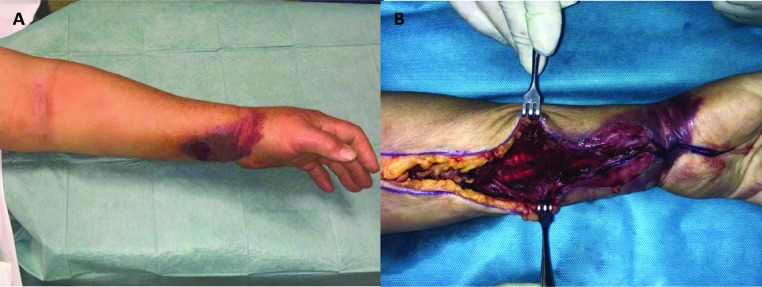
A phlycten on the patient´s left forearm (A); necrotizing fasciitis submitted to surgical debridement and fasciotomy (B)

A 74-year-old female with diabetes mellitus type 2 was admitted to the emergency department due to fever, severe pain and oedema in her left forearm, with two days of evolution, after she has done gardening, but no clear history of trauma. At clinical examination, the patient presented hypotension, fever and a hematic phlycten on her left forearm, which was tense to palpation (Figure 1). Laboratory investigation revealed an elevated C-reactive protein of 386 mg/L, creatine kinase of 13616 U/L and myoglobin of 3269 ug/dL. In addition, acute kidney injury co-occurred with creatinine 2.07 mg/dL and arterial lactate 3.11 mmol/L.  Fluid therapy and empirical antibiotic were initiated with piperacillin/tazobactam, clindamycin and vancomycin, after blood cultures had been collected, and surgical debridement and fasciotomy were performed (Figure 2).  After the surgery, the patient was admitted to the Intensive Care Unit (ICU) with evident deterioration and haemodynamic instability. She maintained intubated and was initiated vasopressor support in high doses. The patient provided her written informed consent for presenting the case and the images. 

Blood cultures identified *Streptococcus pyogenes* as causal pathogen. This confirmed the suspicion of streptococcal toxic shock syndrome (STSS) with necrotizing fasciitis given the persistent hemodynamic instability. As the criteria for STSS were met, clindamycin was maintained for 13 days and concomitant intravenous immunoglobulin was also administered during 3 of these days. Piperacillin/tazobactam and vancomycin were substituted by penicillin G according to *Streptococcus pyogenes* antibiotic susceptibility test. The patient died 13 days after the admission to UCI due to multiple organ dysfunction. 

STSS is an acute and severe life-threatening illness associated with invasive infections by streptococci, mainly group A streptococcus (GAS) [1]. It represents the most fulminant expression of a spectrum of diseases caused by GAS [1] and in spite of medical progresses in the patient care, this condition remains associated with high mortality [2]. The diagnosis of STSS is confirmed when GAS are cultured from normally sterile body fluids in patients with shock and multi-organ failure [2,3]. The exact mechanism of STSS is not fully understood but it is connected the complex interplay between host immunity and pathogen virulence, with the ability of streptococcal toxins to act as superantigen and the host response to streptococcal infection [2]. 

The admission to the ICU and the initiation of supportive treatment of several dysfunction organs is usually necessary. In case of necrotizing fasciitis, aggressive surgical debridement of infected and necrotic tissues associated with systemic antibiotic therapy should be given rapidly, involving high doses of parental beta-lactams, including penicillin G, an agent which is considered first line therapy, in addition to clindamycin for its toxin-neutralising effect [2]. In patients with no clinical response, immunoglobulin is an option, but this indication should be discussed on a case-by-case basis [4]. In our case, the clinical management included medical supervision by a multidisciplinary team of intensive care specialists, plastic surgeon’s specialists and general surgeons. Unfortunately, the patient died 13 days after the admission to UCI due to multiple organ dysfunction. 

## Conflicts of Interest:

None declared.

## References

[1] Lappin E, Ferguson AJ (2009). Gram-positive toxic shock syndromes. Lancet Infect Dis.

[2] Schmitz M, Roux X, Huttner B, Pugin J (2018). Streptococcal toxic shock syndrome in the intensive care unit. Ann Intensive Care.

[3] Wahab A, Nasir B (2018). Streptococcal toxic shock syndrome with primary group A streptococcus peritonitis in a healthy female. J Community Hosp Intern Med Perspect.

[4] Note S, Soentjens P, Van Laer M, Meert P, Vanbrabant P (2018). Streptococcal toxic shock syndrome in a returning traveller. Acta Clin Belg.

